# A Nature-Inspired Solution for Water Management in a Zero-Gap CO_2_ Electrolyzer

**DOI:** 10.1021/acsenergylett.5c01243

**Published:** 2025-06-06

**Authors:** Linlin Xu, Panagiotis Trogadas, Yang Lan, Shuxian Jiang, Shangwei Zhou, Francesco Iacoviello, Wenjia Du, Rhodri Jervis, Marc-Olivier Coppens

**Affiliations:** † Centre for Nature-Inspired Engineering, Department of Chemical Engineering, 4919 University College London, London WC1E 7JE, United Kingdom; ‡ Department of Chemistry, Aristotle University of Thessaloniki, Thessaloniki 54124, Greece; § Electrochemical Innovation Lab, Department of Chemical Engineering, 4919 University College London, London WC1E 7JE, United Kingdom; ∥ Department of Engineering Science, 6396 University of Oxford, Oxford OX1 3PJ, United Kingdom

## Abstract

Electroreduction of carbon dioxide (CO_2_RR) holds great promise as a CO_2_ emission mitigation strategy while producing valuable chemicals. This study draws inspiration from desert-dwelling lizards to design a flow-field that increases the performance of the CO_2_RR in a zero-gap CO_2_ electrolyzer. It achieves a CO partial current density of 165.5 mA cm^–2^ at 200 mA cm^–2^, surpassing those of conventional parallel and serpentine flow-field designs. Unlike more complex strategies that can only partially prevent water flooding or salt precipitation, our approach achieves both, solely by modifying the cathodic flow-field, while using commercial electrocatalysts, membranes, and standard operating conditions. When doubling the cell size, the lizard-inspired serpentine flow-field significantly boosts CO production: CO selectivity is 46% and 97% higher than for a conventional serpentine flow-field at 350 mA cm^–2^ and 400 mA cm^–2^, respectively. Thus, lizard-inspired flow-field technology could provide a step-change in stable, scalable CO_2_RR, even using commercially available components for the use of CO_2_ electrolyzers.

## Graphical abstract

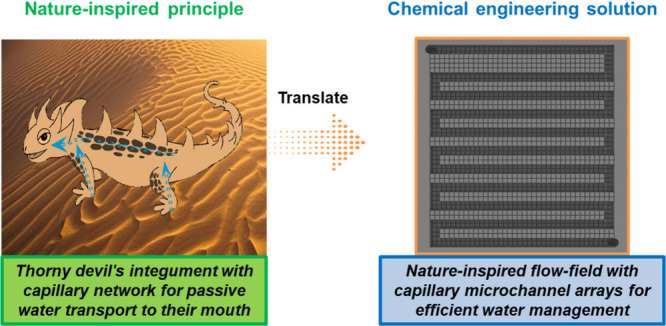

In recent years, there has been growing interest in utilizing renewable energy to drive the electroreduction of carbon dioxide (CO_2_RR), aiming to close the artificial carbon cycle and produce valuable chemicals and fuels.^1–3^ The incorporation of gas diffusion layers (GDLs) and a membrane electrode assembly (MEA) in the cell architecture of zero-gap CO_2_ electrolyzers allows this device to reach industrially relevant current densities (>200 mA cm^–2^) and selective conversion. GDLs enable high current densities by decreasing the diffusion length of CO_2_ from the gas phase to the surface of the MEA where catalysis occurs.^4^ However, the alkaline environment of this device promotes a pathway toward the formation of salt deposits onto the GDLs via the production of carbonates, which impedes CO_2_ transport to catalytic sites and enhances flooding of the cell, resulting in device failure.^4–10^ Strategies to alleviate flooding and salt precipitation in zero-gap CO_2_ electrolyzers with gas-fed cathodes include (i) modification of the concentration and composition of the electrolyte,^11–15^ (ii) alteration of the properties of its cell components such as the GDL and the membrane,^5,16–20^ (iii) pulsed electrolysis,^21–24^ and (iv) optimization of the operating conditions.^4,8,25–28^ An additional strategy to circumvent the flooding issue in such electrolyzers is proper modification of the flow-field design, which is a critical but elusive aspect.

Flow-field design significantly affects the reactant distribution and mass transport within the electrolyzer.^29^ A well-designed flow-field ensures uniform delivery of CO_2_ to the catalyst surface, which enhances its accessibility to catalytic sites, improving the selectivity and yield of the desired products. Recent studies have underscored the significance of flow-field optimization in enhancing CO_2_ electrolyzer performance.^18,30^ For instance, interdigitated flow-fields exhibit ∼45 mA cm^–2^ higher CO_2_RR current density than their serpentine counterparts at a gas flow rate of 6 sccm, indicating more efficient transport of gaseous CO_2_ to the catalyst (Cu/C/PTFE).^31^ The pressure drop on the cathode side of the flow-field plays a critical role in the stable operation of zero-gap CO_2_ electrolyzers, as it enhances CO_2_ transport through the GDL and around salt and water blockages.^5^ In a study using a small-scale cell size of 5.06 cm^2^, serpentine flow-fields have the highest pressure drop (∼ 143 Pa), which is ∼81% and ∼143% higher than the interdigitated and parallel flow-fields, respectively, and they demonstrate the highest CO partial current density (∼205 mA cm^–2^) at 2.76 V.^5^ Even though these results are promising at small scale, serpentine flow-fields are characterized by excessive pressure drop at larger scale (>25 cm^2^),^32^ and thus, the engineering of alternate flow-field designs preventing flooding and salt precipitation is necessary. Preliminary results demonstrate that a 50 cm^2^ spiral flow-field with unidirectional and uniform distribution (UDF) enhances the mass transport of CO_2_ to the surface of the MEA and, thus, the production rate of CO maintaining a low pressure drop (∼ 6 kPa).^33^

Herein, we employ our nature-inspired chemical engineering (NICE) methodology^32,34–37^ to design new flow-fields for zero-gap CO_2_ electrolyzers, boosting their efficiency via optimized structural features of the flow-fields (Figure 1). The NICE approach is based on the fundamental understanding of the mechanisms underpinning desired properties in biological organisms and their implementation in technological applications, without neglecting the differences in context between nature and technology.^32,34–37^ The flow-field design for CO_2_ electrolyzers herein draws inspiration from desert-dwelling lizards, such as the Australian thorny devil and Texan horned lizard, which possess intricate networks of capillary channels in their integument that facilitate passive water transport.^36,37^ The lizard-inspired flow-field is designed to improve water management and enhance mass transport in electrochemical devices;^36,37^ recently, it has been implemented into polymer electrolyte membrane fuel cells (PEMFCs) showcasing a stable, flood-free operation at 100% relative humidity (RH) and scalability.^36,37^ These results suggest that such a lizard-inspired design might also be a promising candidate for the elimination of water flooding and salt precipitation in zero-gap CO_2_ electrolyzers. The capillary-driven water transport mechanism used by lizards is implemented by creating capillaries directly on the surface of conventional flow-fields in a zero-gap CO_2_ electrolyzer. The water transport mechanism, driven by capillary action in the desert-dwelling lizards’ integument, remains effective when scaled up, as confirmed by quantitative analysis showing that capillary forces dominate over gravitational and viscous forces.^37^ In our design, hydrophilic capillaries are laser-engraved directly onto the surface of a serpentine flow-field (Section S1.3), which is widely used due to its higher pressure drop compared to a parallel flow-field, allowing the continuous transport of reactants through the GDL to the electrocatalyst and ensuring good catalyst utilization.^5,38^ The positive impact of this design on the wettability of the surface of the flow-field was initially demonstrated via contact angle (CA) measurements. A water droplet (∼60 °CA) on the surface of a lizard-inspired serpentine flow-field rapidly permeates through its structure (Video S1), whereas it remains stagnant (∼90 °CA) on the surface of a conventional serpentine flow-field, demonstrating that this nature-inspired design effectively enhances passive water transport in the flow-field. Based on these initial promising results, we compare the efficiency and operational stability of a lizard-inspired serpentine flow-field against conventional parallel and serpentine flow-field based CO_2_ electrolyzers. X-ray microcomputed tomography (micro-CT) is utilized to gain deeper insights into the electrochemical and structural dynamics within the different flow-fields.

**Figure 1:**
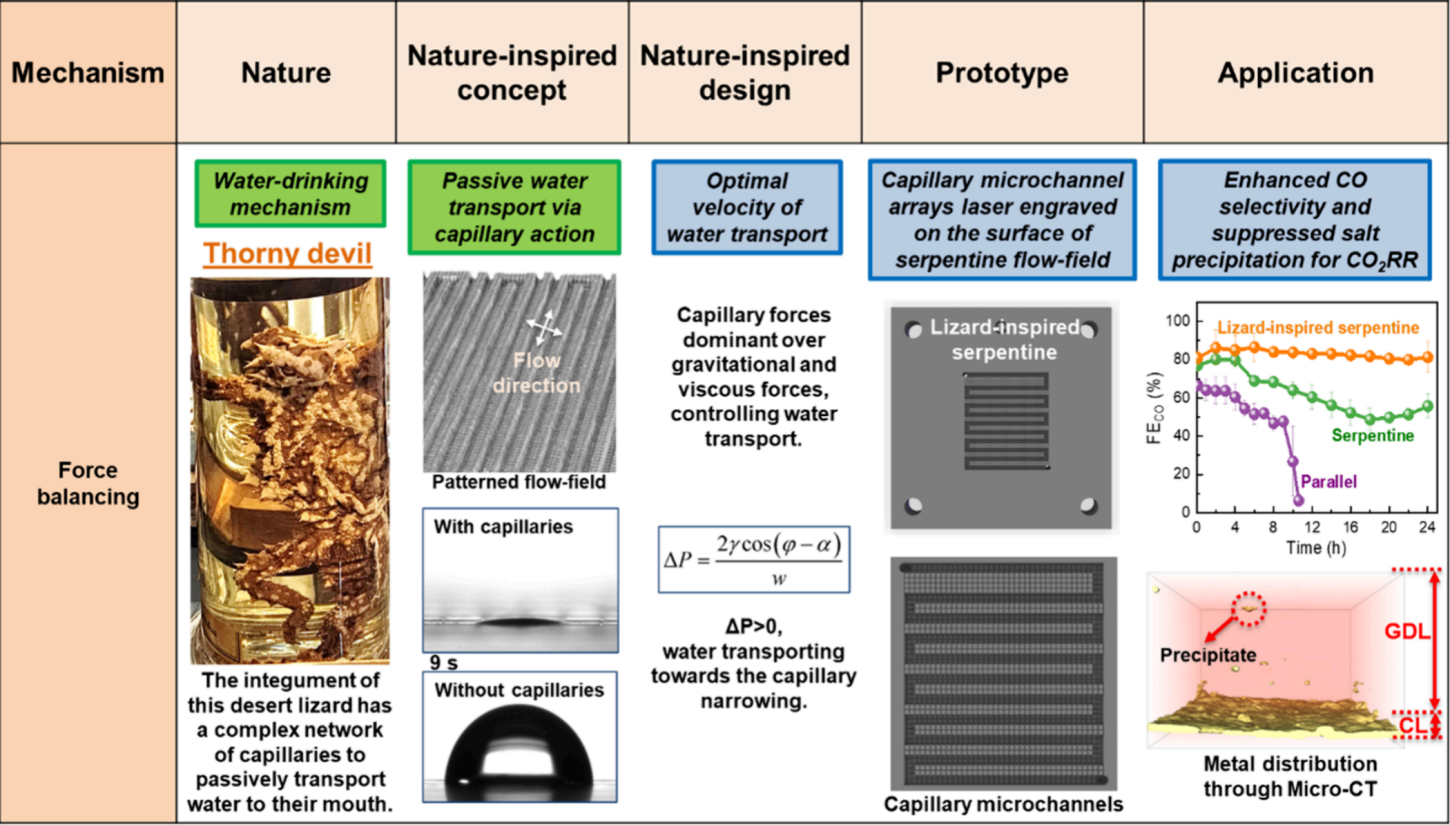
Implementation of the NICE approach in the design and engineering of a flow-field for a zero-gap CO_2_ electrolyzer, inspired by desert-dwelling lizards. The image of the thorny devil is taken from a sample at the UCL Grant Museum of Zoology, London, UK.

Three distinct flow-fields (parallel, serpentine, and lizard-inspired serpentine) were employed at the cathode side of a zero-gap CO_2_ electrolyzer to evaluate changes in activity and selectivity of CO_2_RR on an Ag gas diffusion electrode (GDE) at room temperature and atmospheric pressure. CO_2_RR was conducted at discrete current densities: 20, 50, 100, 150, 200, 250, and 300 mA cm^–2^, with each point held for 20 min under steady-state conditions on electrodes with a geometric area of 2.25 cm^2^ (Figure 2). At lower current densities (20–150 mA cm^–2^), the CO_2_RR exhibits similar cell voltages for all flow-fields (Figure 2a), indicating that CO_2_ can then reach all catalytic sites unobstructed. At 200 mA cm^–2^, the CO partial current density of parallel and serpentine flow-fields remain similar, but both are surpassed by the lizard-inspired serpentine flow-field, which achieves a maximum CO partial current density of ∼165.5 mA cm^–2^ (Figure 2b). At 250 mA cm^–2^, the lizard-inspired serpentine flow-field outperforms the serpentine and parallel flow-fields by ∼14.4% and ∼38.7%, respectively, while at 300 mA cm^–2^, it shows a ∼21.4% and ∼69.8% improvement over the serpentine and parallel flow-fields, respectively. This trend is also reflected in the energy efficiency of the CO production (Figure 2c). Selectivity is also dependent on the chosen flow-field and applied current density. For all flow-fields tested, CO selectivity decreases as current density increases. At 300 mA cm^–2^, the Faradaic efficiency (FE) of H_2_ in parallel, serpentine, and lizard-inspired serpentine flow-field based CO_2_ electrolyzers reach ∼59%, ∼39%, and ∼31%, respectively (Figure 2d), suggesting mass transport limitations arise, lowering CO_2_ utilization and favoring the hydrogen evolution reaction (HER).^5^

**Figure 2:**
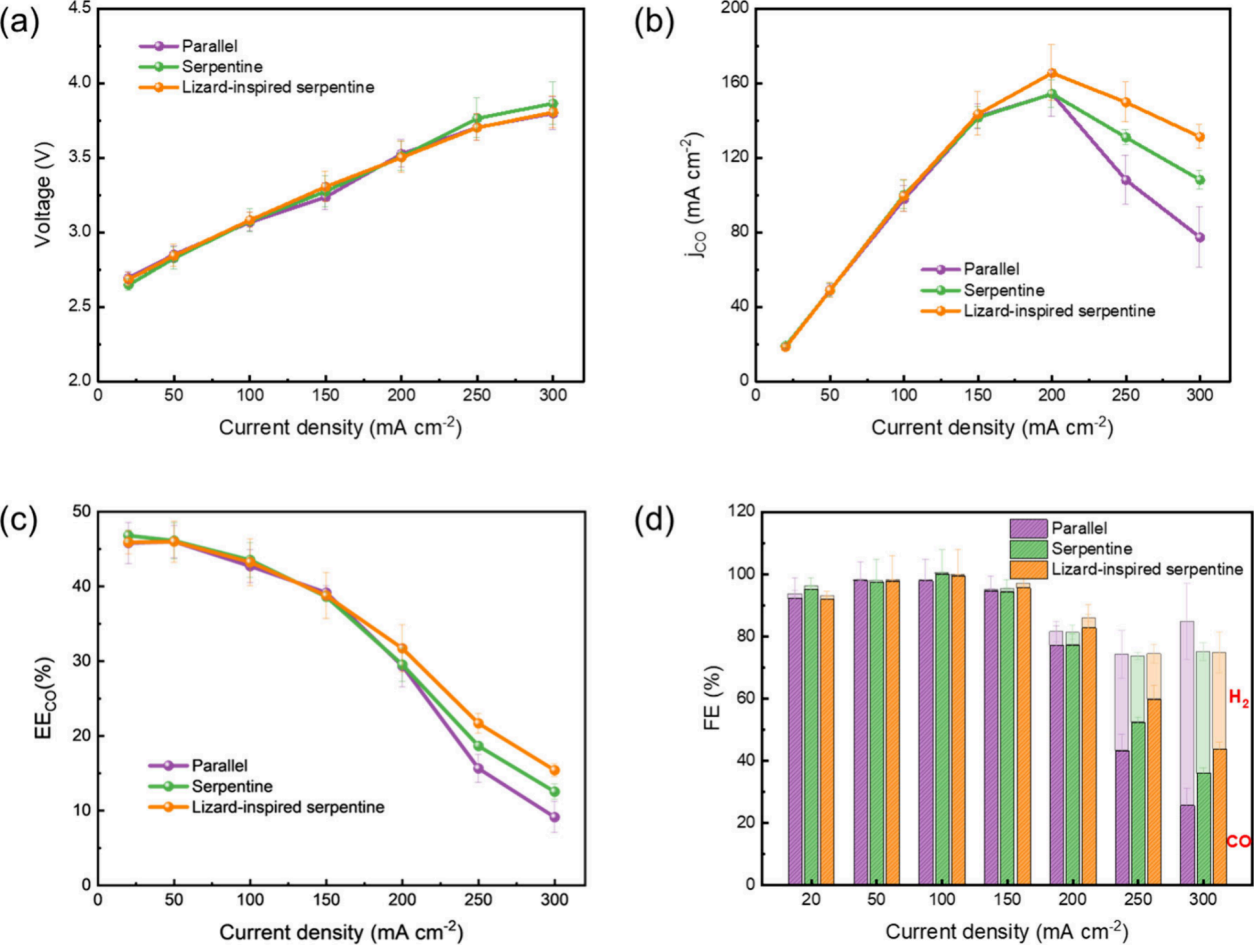
(a) Cell voltage, (b) average partial current density of CO, (c) energy efficiency of CO production, and (d) total Faradaic efficiency (CO-bottom bars, H_2_-top bars) for parallel, serpentine, and lizard-inspired serpentine flow-field based CO_2_ electrolyzers. Data were collected at discrete current densities (20, 50, 100, 150, 200, 250, and 300 mA cm^–2^). Each experiment is replicated across three independent measurements, and the error bars represent the standard deviation. The geometric area of each flow-field is 2.25 cm^2^.

To gain further insights into the observed phenomena, electrochemical impedance spectroscopy was performed (Figure S4). High-frequency resistance (HFR, or Ohmic resistance) and total charge transfer resistance of the cell are derived from equivalent circuit modeling (Figure S4a).^39^ The HFR for all flow-fields remains relatively constant, between 0.5 and 1.0 Ω cm^2^, due to employing the same electrolyte (Figure S4c). Analysis of the HFR suggests that the increase in voltage (e.g., ∼3.76 V for serpentine vs ∼3.70 V for a lizard-inspired serpentine flow-field at 250 mA cm^–2^ in Figure 2a) is attributed to a higher HFR (e.g., ∼0.71 Ω cm^2^ for serpentine vs ∼0.45 Ω cm^2^ for a lizard-inspired serpentine flow-field at 250 mA cm^–2^ in Figure S4c) and potential degradation of the membrane, exacerbated at higher current densities.^39,40^ The disparity in FE_CO_ between serpentine and lizard-inspired serpentine flow-fields (Figure 2d) is likely due to mechanical stress on the membrane during electrolysis, which is an additional result of membrane dehydration.^39^ Figure S4b illustrates more significant differences in the Nyquist plots among the different flow-fields, particularly at low frequencies. The low-frequency resistance dominates at low current densities but decreases significantly with increasing current density, suggesting that a substantial portion is related to charge transfer resistance (Figure S4d).^39^ The lower charge transfer resistance of a lizard-inspired serpentine flow-field compared to a serpentine flow-field at higher current densities reflects enhanced charge transferability, and reduced polarization losses during CO_2_RR.^41,42^

To evaluate the stability of these zero-gap CO_2_ electrolyzers, the CO_2_RR was conducted over 24 h at 200 mA cm^–2^ (Figure 3). The cell voltage of all flow-fields initially decreases slightly during the first 2 h (Figure 3a). As the CO_2_RR progresses, only the lizard-inspired serpentine flow-field maintains a stable cell voltage (∼3.5 V) and CO selectivity (∼81.3%) throughout the operation of the device (Figure 3b), indicating that CO_2_ has access to the catalytic sites of the MEA and, thus, flooding is avoided. On the contrary, in the case of parallel and serpentine flow-fields, there is a gradual increase in cell voltage accompanied by a decrease in CO selectivity. After 10 h of operation, there is a sudden drop of the cell voltage for the parallel flow-field, coinciding with a drastic reduction in CO selectivity to ∼6.4% due to salt precipitation (Figure 3d) and flooding, boosting H_2_ selectivity to ∼50% (Figure 3c). The parallel flow-field fails to operate past this point. For the serpentine flow-field, the cell voltage increases steadily over the first 22 h and then decreases, suggesting local membrane-electrode delamination or electrolyte breakthrough, likely induced by severe salt accumulation. This voltage collapse is accompanied by a reduction in CO selectivity of ∼ 21% after 24 h of operation, along with increased H_2_ selectivity (∼20%), consistent with performance losses due to flooding and salt blockage, which is clearly visible on the back of the cathode side of the Ag GDEs post electrolysis (Figure 3d).

**Figure 3:**
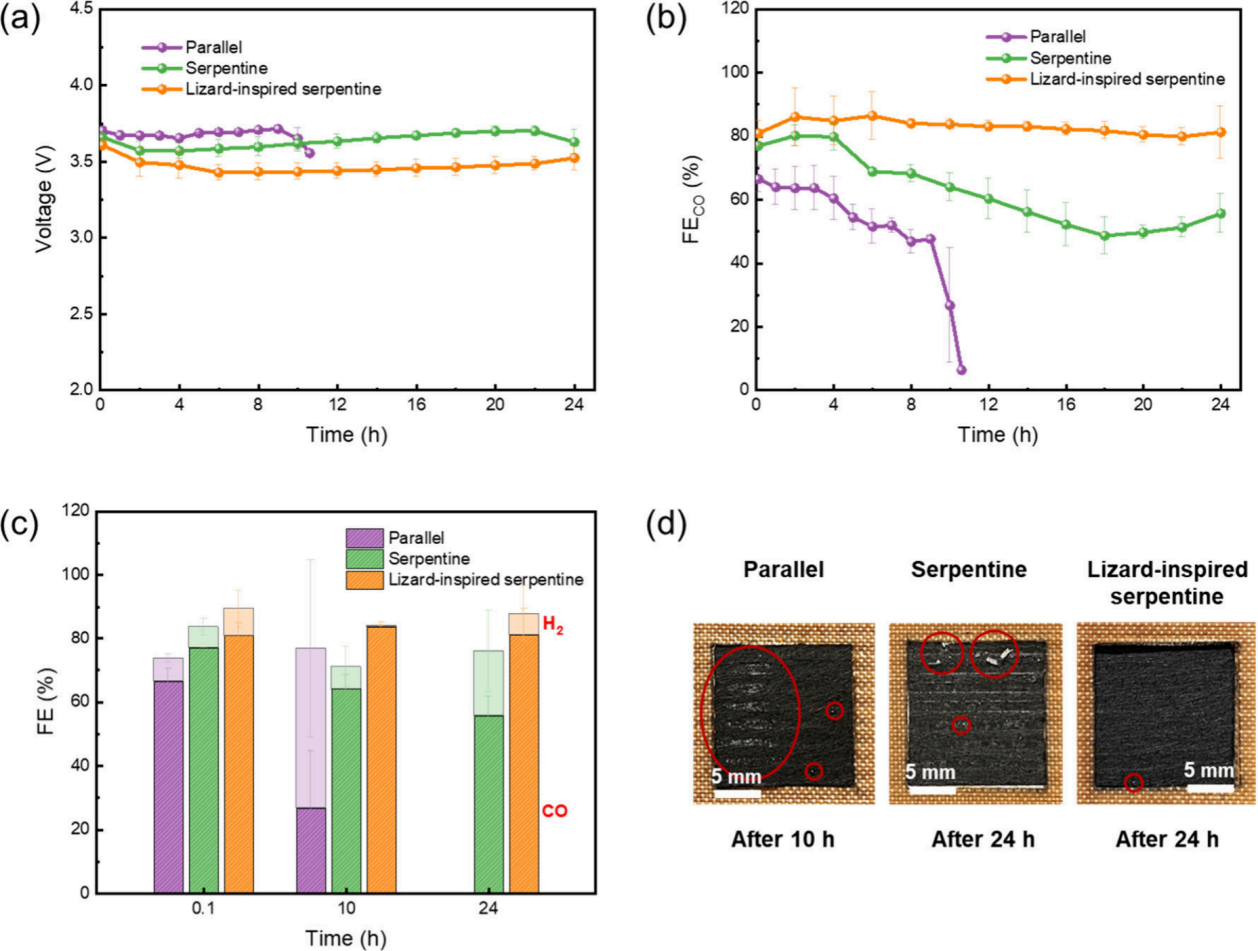
Variation of (a) cell voltage, (b) Faradaic efficiency of CO, and (c) total Faradaic efficiency over time during the CO_2_RR. (d) Images of the back of the cathode side of Ag GDEs after electrolysis at 200 mA cm^–2^, showing the precipitation of salt crystals (red circle). The parallel flow-field based CO_2_ electrolyzer fails to operate after 10 h due to salt precipitation and flooding. Each experiment is replicated across three independent measurements, and the error bars represent the standard deviation. The geometric area of each flow-field is 2.25 cm^2^.

XRD measurements (Figure S5) of Ag GDEs show that the crystallographic planes of Ag remain unchanged after CO_2_ electrolysis,^43^ while the emergence of additional peaks (marked with blue diamonds) indicates the formation of KHCO_3_ during the reaction of hydrogen peroxide, a byproduct of CO_2_RR, with the anolyte.^8^ The presence of Ag and K on Ag GDEs is also confirmed by SEM (Figure S6).

The formation of salt precipitates within the GDE poses a significant challenge to the stable operation of zero-gap electrolyzers with an alkaline anolyte. A higher concentration of K^+^ leads to a reduction in FE_CO_ due to an increase in the electro-osmotic drag of water, exacerbating flooding of the cathode and resulting in increased mass transport limitations for CO_2_RR.^7^ Micro-CT imaging was employed to quantitatively evaluate the salt precipitation on all flow-fields after durability measurement (Figure S7). The minimal Ag presence in the fresh GDE is due to the initial infiltration of Ag nanoparticles during electrode preparation. In contrast, increased brightness is highlighted in the GDLs post electrolysis, indicating localized accumulation of K, likely resulting from uneven distribution of reactants. However, compared to the lizard-inspired serpentine flow-field with only few bright spots, the parallel and serpentine flow-fields show widespread and uneven accumulation of K.

A field-of-view of 300 × 300 × 200 voxel in *x*-*y*-*z* orientation, with an isotropic voxel size of approximately 1.78 μm, was selected for material segmentation of micro-CT images (Figure S8). Given the nanoscale size of Ag, distinguishing it accurately from K proves to be challenging. Therefore, both Ag and K are categorized as “metals” in Figure S8b. However, considering that the unreacted GDL contains minimal Ag, it is inferred that the metallic content observed in the reacted GDLs predominantly consists of potassium precipitates formed during electrolysis. A relatively loose layer of dispersed Ag particles is present on fresh GDE (Figure 4a). Notably, a significantly higher number of precipitates is observed within the GDEs mounted on parallel and serpentine flow-fields compared to the GDE on lizard-inspired serpentine flow-field, suggesting greater potassium salt accumulation in the GDL pores, which impedes CO_2_ transport to the catalytic sites (Figure 4a).^44^ The GDL on the serpentine flow-field contains the highest amount of salt precipitates (Figure 4b-c), with their quantities in the GDLs for the parallel, serpentine, and lizard-inspired serpentine flow-fields being ∼149, 377, and 72, respectively. Specifically, at a depth between 100–200 μm, the metal fraction in the Ag GDE of the serpentine flow-field reaches 6%–10%, while in the lizard-inspired serpentine flow-field it remains below 1%. Even though the parallel flow-field operates flood-free for 10 h, severe salt precipitation and significant flooding occurs thereafter, as indicated by the faults observed in the GDL (Figure S8b, Figure S9b). Additionally, the lizard-inspired serpentine flow-field shows significantly fewer large precipitates within the GDL compared to the parallel and serpentine flow fields (Figure 4c). A histogram of the size distribution of metal (Ag or Ag and K) within the GDL (depth: 100–350 μm) of these flow-fields was constructed, revealing that the number of precipitates larger than 1000 μm^3^ is ∼19 in the parallel flow-field, ∼36 in the serpentine flow-field, and only ∼6 in the lizard-inspired serpentine flow-field (Figure 4c). These observations indicate increased potassium salt precipitation in parallel and serpentine flow-fields, correlating with their degradation in FE_CO_. Therefore, upon the incorporation of the lizard-inspired capillary microchannels onto the conventional serpentine flow-field, the amount of salt is minimized due to efficient water management.

**Figure 4:**
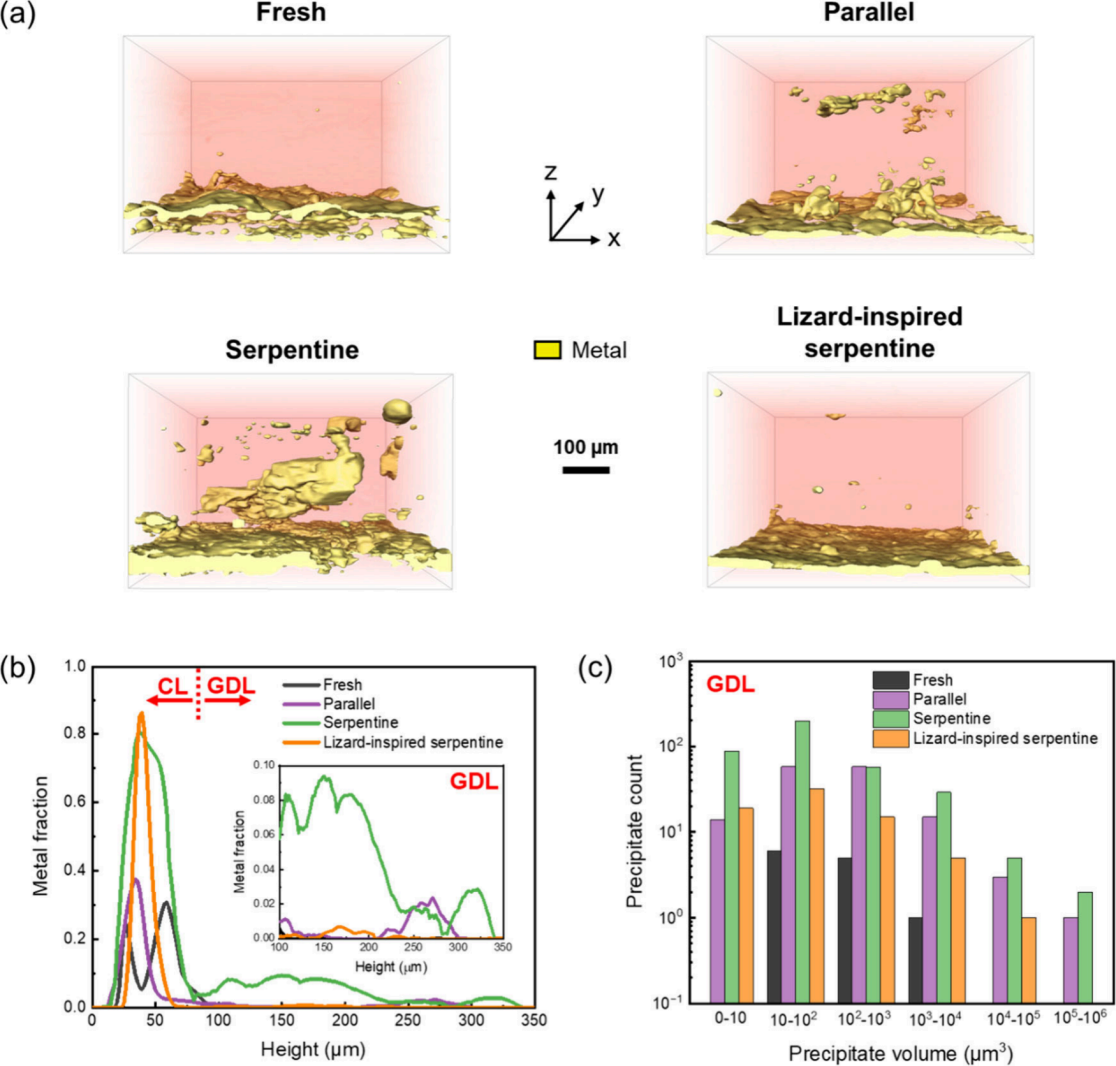
(a) Three-dimensional visualization of metal before and after electrolysis captured by micro-CT. The metal (labeled in yellow) denotes Ag for the GDE before reaction and Ag+K for the GDEs after reaction. (b) The metal fraction in Ag GDEs (depth: 0–350 μm), with the inset showing the metal fraction in GDLs (depth: 100–350 μm). (c) Histogram of the size distribution of Ag or Ag+K within the GDLs (depth: 100–350 μm). This is a statistical analysis of the volume of all precipitates within the GDLs, with the horizontal axis representing the volume of each precipitate and the vertical axis representing the number of precipitates within that volume range. The geometric area of each flow-field is 2.25 cm^2^.

These promising results are obtained when capillary microchannels are incorporated within a flow-field for a geometric MEA area of 2.25 cm^2^. At such small scale, the distribution of CO_2_ within the flow-field and the removal of products are inherently more uniform.^33^ However, as the size of the electrolyzer increases and higher conversion efficiency is targeted, spatial variations in reactant distribution along the gas channel become more significant.^5^ To evaluate the effect of a lizard-inspired serpentine flow-field design in a larger electrolyzer, we doubled the cell size from 2.25 to 5.06 cm^2^ (Figure S10). This upscaling was performed to test the robustness of the flow-field design under increased spatial constraints. The lizard-inspired serpentine flow-field retains its properties effectively, maintaining a higher CO selectivity and reduced H_2_ formation at current densities exceeding 200 mA cm^–2^ (Figure S10a-c). At 300 mA cm^–2^, the CO selectivity of the lizard-inspired serpentine flow-field is 21% and 16% higher than that of the serpentine flow-field at cell sizes of 2.25 cm^2^ and 5.06 cm^2^, respectively. Furthermore, at current densities of 350 mA cm^–2^ and 400 mA cm^–2^, the CO selectivity exceeds that of the traditional serpentine flow-field by 46% and 97%, respectively, for a cell size of 5.06 cm^2^. The lizard-inspired design enhances gas flow management and mass transport dynamics, ensuring efficient removal of salt byproducts and water.^36,37^ The lizard-inspired serpentine flow-field (5.06 cm^2^) still has a minimal amount of salt precipitate (Figure S11c), while high salt precipitation on the back of the cathode side of the Ag GDE is observed for the parallel flow-field, where the channels are flooded (Figure S11a). This improvement is critical for sustaining a high CO_2_RR efficiency and selectivity in larger devices. Moreover, the lizard-inspired serpentine flow-field achieves these enhancements without incurring additional voltage penalties (Figure S10d). This characteristic is particularly advantageous, as it allows for improved performance and selectivity without compromising the overall energy efficiency of the system.

In summary, this study highlights the significant impact of a nature-inspired flow-field design on the activity and stability of the CO_2_RR in zero-gap electrolyzers, building on its proven success in PEMFCs. The comparative analysis of parallel, serpentine, and lizard-inspired serpentine flow-field based CO_2_ electrolyzers reveals that while all designs exhibit comparable performance at lower current densities, significant differences arise at higher current densities. Specifically, at 200 mA cm^–2^, the lizard-inspired serpentine flow-field achieves a maximum partial current density for CO of 165.5 mA cm^–2^, outperforming both the parallel and serpentine flow-fields. This indicates that the lizard-inspired design is more effective in overcoming mass transport limitations and sustaining the CO selectivity under high current densities. Furthermore, the lizard-inspired serpentine flow-field shows superior stability, maintaining a stable cell voltage and a CO selectivity of approximately 81.3% over 24 h at 200 mA cm^–2^. In contrast, the conventional serpentine flow-field experiences a decrease in CO selectivity, although it is less severe than the parallel flow-field, which suffers a sharp decline in CO selectivity to 6.4% after just 10 h. These variations are largely attributed to the effective management of water and reduced salt precipitation afforded by the lizard-inspired design. Micro-CT analysis confirms that the lizard-inspired serpentine flow-field has minimal salt crystal formation, which prevents the blockage of the active sites of the catalyst and gas transport channels, further underscoring its potential for enhancing CO_2_RR performance.

The observed salt precipitation detailed in the above sections contradicts earlier reports in the literature suggesting that a low concentration of the anolyte combined with an anion exchange membrane-based MEA can avoid salt precipitation.^8,45^ Even an anolyte containing 0.1 M KHCO_3_ combined with a Sustainion-based MEA leads to significant salt concentration after short operation of the CO_2_ electrolyzer.

While the field of CO_2_RR has advanced significantly in catalyst development and optimization of operating conditions, flow field design remains an underrepresented area despite its critical influence on performance and scalability of the CO_2_ electrolyzer, as evidenced by the limited number of publications on this topic. A recent report investigated the uniform distribution of CO_2_ across the catalyst layer via a multiserpentine flow field design achieving 12 h of stable operation at 100 mA cm^–2^.^46^ However, the optimization of gas flow alone within the flow field is insufficient; our study goes beyond the uniformity of the CO_2_ distribution, directly addressing critical challenges such as water flooding and salt precipitation through a nature-inspired flow field design achieving superior stability with 24 h of operation at 200 mA cm^–2^. Another report investigates the influence of operating parameters, namely anolyte concentration, cation species, membrane thickness, and temperature, on cation accumulation and salt formation.^47^ The combination of optimal parameters results in a 144 h stable operation at 200 mA cm^–2^ with no measurable salt deposition. However, under the same operating conditions as in our study (0.1 M KHCO_3_, room temperature, 50 μm thick Sustainion membrane, 200 mA cm^–2^), the operation of the device fails after only 100 min. In contrast, our work employs a single, practical strategy that effectively prevents water flooding and salt accumulation without relying on complex parameter combinations, resulting in a stable 24 h operation at 200 mA cm^–2^.

Thus, none of the other proposed strategies (addition of solvents to the cathode to dissolve and remove precipitates, rinsing of the cell, modified polymer membranes, optimization of the operating conditions) have emerged as a single standalone solution to this issue. They are always combined, e.g. cell rinsing and optimal operating conditions, to enable long-term operation, hereby increasing the complexity of the electrolyzer setup and the electrochemical measurements.^4,8,47,48^ On the contrary, our nature-inspired approach circumvents water flooding and salt precipitation on the GDE via sole modification of the cathodic flow-field. Commercial electrocatalysts and membranes are used for the preparation of GDE, while common operating conditions are utilized. This reduces the cost and complexity of the CO_2_RR setup allowing the stable operation of the zero-gap electrolyzer.

Future research should focus on several key areas to further enhance the CO_2_RR performance and scalability. Increasing the cell size beyond the tested 5.06 cm^2^ value will help assess the effectiveness for larger, industrially relevant systems. Additionally, optimizing operational parameters such as flow rate, anolyte composition and concentration, temperature, pressure, as well as pulsed electrolysis—an area that remains scarcely explored in current literature—could further improve performance by better preventing the formation of carbonate salt. Moreover, extending stability tests beyond 24 h will be essential to validate long-term durability under practical conditions. Such studies could incorporate periodic electrochemical diagnostics and post-mortem structural analysis to monitor degradation phenomena over time. Furthermore, integrating computational fluid dynamics (CFD) simulations to elucidate the impact of flow field design on CO_2_ transport and salt/water blockages could bridge gaps in mechanistic understanding and guide the rational design of the CO_2_RR systems.

## Supplementary Material





## Notes

Experimental section including materials, electrode preparation and membrane pretreatment, flow-field fabrication (Figure S1), CO_2_RR electrolyzer components (Figure S2), CO_2_RR setup (Figure S3), electrochemical testing, product analysis, and sample characterization; equivalent circuit model used for fitting of the EIS data, and analysis via Nyquist plots, HFR and total charge transfer resistance (Figure S4); XRD pattern of Ag catalyst before and after CO_2_RR (Figure S5); SEM images and EDS mapping of Ag GDEs (Figure S6); Micro-CT images of Ag GDEs (Figure S7); The schematic diagram of material segmentation (Figure S8); Material fraction of cathode Ag GDEs (Figure S9); Performance comparison of CO_2_ electrolyzers with different flow-fields (Figure S10); Images of the back of cathode Ag GDEs and cathode flow-fields after electrolysis (Figure S11) (PDF)
Movie S1: a water droplet (∼ 60 °CA) on the surface of a lizard-inspired serpentine flow-field rapidly permeates through its structure (MP4)

## References

[1] Kibria M. G., Edwards J. P., Gabardo C. M., Dinh C.-T., Seifitokaldani A., Sinton D., Sargent E. H. (2019). Electrochemical CO_2_ Reduction
into Chemical Feedstocks: From Mechanistic Electrocatalysis Models
to System Design. Adv. Mater..

[2] Chen J., Wang L. (2022). Effects of the Catalyst
Dynamic Changes and Influence of the Reaction
Environment on the Performance of Electrochemical CO_2_ Reduction. Adv. Mater..

[3] Farooqi S. A., Farooqi A. S., Sajjad S., Yan C., Victor A. B. (2023). Electrochemical
Reduction of Carbon Dioxide into Valuable Chemicals: A Review. Environ. Chem. Lett..

[4] Sassenburg M., Kelly M., Subramanian S., Smith W. A., Burdyny T. (2023). Zero-Gap Electrochemical
CO_2_ Reduction Cells: Challenges and Operational Strategies
for Prevention of Salt Precipitation. ACS Energy
Lett..

[5] Subramanian S., Yang K., Li M., Sassenburg M., Abdinejad M., Irtem E., Middelkoop J., Burdyny T. (2023). Geometric Catalyst Utilization in Zero-Gap CO_2_ Electrolyzers. ACS Energy Lett..

[6] Kong Y., Liu M., Hu H., Hou Y., Vesztergom S., Gálvez-Vázquez M. de J., Zelocualtecatl Montiel I., Kolivoška V., Broekmann P. (2022). Cracks as Efficient Tools to Mitigate
Flooding in Gas Diffusion Electrodes Used for the Electrochemical
Reduction of Carbon Dioxide. *Small*. Methods.

[7] Mardle P., Cassegrain S., Habibzadeh F., Shi Z., Holdcroft S. (2021). Carbonate
Ion Crossover in Zero-Gap, KOH Anolyte CO_2_ Electrolysis. J. Phys. Chem. C.

[8] Disch J., Bohn L., Metzler L., Vierrath S. (2023). Strategies for the
Mitigation of Salt Precipitation in Zero-Gap CO_2_ Electrolyzers
Producing CO. J. Mater. Chem. A.

[9] Rabinowitz J. A., Kanan M. W. (2020). The Future
of Low-Temperature Carbon Dioxide Electrolysis
Depends on Solving One Basic Problem. Nat. Commun..

[10] O’Brien C. P., Miao R. K., Shayesteh
Zeraati A., Lee G., Sargent E. H., Sinton D. (2024). CO_2_ Electrolyzers. Chem. Rev..

[11] Gao D., Arán-Ais R. M., Jeon H. S., Roldan Cuenya B. (2019). Rational Catalyst
and Electrolyte Design for CO_2_ Electroreduction towards
Multicarbon Products. Nat. Catal..

[12] Xu A., Govindarajan N., Kastlunger G., Vijay S., Chan K. (2022). Theories for
Electrolyte Effects in CO_2_ Electroreduction. Acc. Chem. Res..

[13] Endrődi B., Samu A., Kecsenovity E., Halmágyi T., Sebők D., Janáky C. (2021). Operando Cathode
Activation with
Alkali Metal Cations for High Current Density Operation of Water-Fed
Zero-Gap Carbon Dioxide Electrolysers. Nat.
Energy.

[14] Yin Z., Peng H., Wei X., Zhou H., Gong J., Huai M., Xiao L., Wang G., Lu J., Zhuang L. (2019). An Alkaline Polymer Electrolyte CO_2_ Electrolyzer
Operated with Pure Water. Energy Environ. Sci..

[15] De
Mot B., Ramdin M., Hereijgers J., Vlugt T. J. H., Breugelmans T. (2020). Direct Water
Injection in Catholyte-Free Zero-Gap Carbon Dioxide Electrolyzers. ChemElectroChem..

[16] Lees E. W., Mowbray B. A. W., Parlane F. G. L., Berlinguette C. P. (2022). Gas Diffusion
Electrodes and Membranes for CO_2_ Reduction Electrolysers. Nat. Rev. Mater..

[17] Wakerley D., Lamaison S., Wicks J., Clemens A., Feaster J., Corral D., Jaffer S. A., Sarkar A., Fontecave M., Duoss E. B., Baker S., Sargent E. H., Jaramillo T. F., Hahn C. (2022). Gas Diffusion Electrodes,
Reactor Designs and Key Metrics of Low-Temperature
CO_2_ Electrolysers. Nat. Energy.

[18] Chen Q., Wang X., Zhou Y., Tan Y., Li H., Fu J., Liu M. (2024). Electrocatalytic CO_2_ Reduction to C_2+_ Products in Flow Cells. Adv. Mater..

[19] Reyes A., Jansonius R. P., Mowbray B. A. W., Cao Y., Wheeler D. G., Chau J., Dvorak D. J., Berlinguette C. P. (2020). Managing
Hydration at the Cathode Enables Efficient CO_2_ Electrolysis
at Commercially Relevant Current Densities. ACS Energy Lett..

[20] Li M., Idros M. N., Wu Y., Burdyny T., Garg S., Zhao X. S., Wang G., Rufford T. E. (2021). The Role of Electrode
Wettability in Electrochemical Reduction of Carbon Dioxide. J. Mater. Chem. A.

[21] Chung Y. L., Kim S., Lee Y., Wijaya D. T., Lee C. W., Jin K., Na J. (2024). Pulsed Electrolysis
for CO_2_ Reduction: Techno-Economic
Perspectives. iScience.

[22] Xu Y., Edwards J. P., Liu S., Miao R. K., Huang J. E., Gabardo C. M., O’Brien C. P., Li J., Sargent E. H., Sinton D. (2021). Self-Cleaning CO_2_ Reduction
Systems: Unsteady
Electrochemical Forcing Enables Stability. ACS
Energy Lett..

[23] DiDomenico R. C., Hanrath T. (2022). Pulse Symmetry Impacts the C_2_ Product Selectivity
in Pulsed Electrochemical CO_2_ Reduction. ACS Energy Lett..

[24] Obasanjo C. A., Zeraati A. S., Shiran H. S., Nguyen T. N., Sadaf S. M., Kibria M. G., Dinh C.-T. (2022). In Situ
Regeneration of Copper Catalysts
for Long-Term Electrochemical CO_2_ Reduction to Multiple
Carbon Products. J. Mater. Chem. A.

[25] Xie K., Ozden A., Miao R. K., Li Y., Sinton D., Sargent E. H. (2022). Eliminating the Need for Anodic Gas
Separation in CO_2_ Electroreduction Systems via Liquid-to-Liquid
Anodic Upgrading. Nat. Commun..

[26] M.
Gabardo C., Seifitokaldani A., P. Edwards J., Dinh C.-T., Burdyny T., Golam Kibria M., P. O’Brien C., H. Sargent E., Sinton D. (2018). Combined High Alkalinity
and Pressurization Enable Efficient CO_2_ Electroreduction
to CO. Energy Environ. Sci..

[27] Ahn S. T., Abu-Baker I., Palmore G. T. R. (2017). Electroreduction of CO_2_ on Polycrystalline Copper: Effect of Temperature on Product Selectivity. Catal. Today.

[28] Samu A. A., Kormányos A., Kecsenovity E., Szilágyi N., Endrődi B., Janáky C. (2022). Intermittent
Operation of CO_2_ Electrolyzers at Industrially Relevant
Current Densities. ACS Energy Lett..

[29] Jung B., Park S., Lim C., Lee W. H., Lim Y., Na J., Lee C.-J., Oh H.-S., Lee U. (2021). Design Methodology
for Mass Transfer-Enhanced Large-Scale Electrochemical Reactor for
CO_2_ Reduction. Chem. Eng. J..

[30] Ma D., Jin T., Xie K., Huang H. (2021). An Overview of Flow Cell Architecture
Design and Optimization for Electrochemical CO_2_ Reduction. J. Mater. Chem. A.

[31] Xing Z., Hu L., Ripatti D. S., Hu X., Feng X. (2021). Enhancing Carbon Dioxide
Gas-Diffusion Electrolysis by Creating a Hydrophobic Catalyst Microenvironment. Nat. Commun..

[32] Trogadas P., Cho J. I. S., Neville T. P., Marquis J., Wu B., Brett D. J. L., Coppens M. O. (2018). A Lung-Inspired Approach to Scalable
and Robust Fuel Cell Design. Energy Environ.
Sci..

[33] Jung B., Park S., Lim C., Lee W. H., Lim Y., Na J., Lee C.-J., Oh H.-S., Lee U. (2021). Design Methodology
for Mass Transfer-Enhanced Large-Scale Electrochemical Reactor for
CO_2_ Reduction. Chem. Eng. J..

[34] Coppens M. O. (2021). Nature-Inspired
Chemical Engineering for Process Intensification. Annu. Rev. Chem. Biomol. Eng..

[35] Trogadas P., Coppens M. O. (2020). Nature-Inspired
Electrocatalysts and Devices for Energy
Conversion. Chem. Soc. Rev..

[36] Xu L., Trogadas P., Zhou S., Jiang S., Wu Y., Rasha L., Kockelmann W., Yang J. D., Neville T., Jervis R., Brett D. J. L., Coppens M.-O. (2024). A Scalable and Robust
Water Management Strategy for PEMFCs: Operando Electrothermal Mapping
and Neutron Imaging Study. Adv. Sci..

[37] Trogadas P., S. Cho J. I., Rasha L., Lu X., Kardjilov N., Markötter H., Manke I., R. Shearing P., L. Brett D. J., Coppens M.-O. (2024). A Nature-Inspired Solution for Water
Management in Flow Fields for Electrochemical Devices. Energy Environ. Sci..

[38] Zhao J. Y., Liu Y., Li W., Wen C. F., Fu H. Q., Yuan H. Y., Liu P. F., Yang H. G. (2023). A Focus on the Electrolyte: Realizing
CO_2_ Electroreduction from Aqueous Solution to Pure Water. Chem. Catalysis.

[39] Mardle P., Gangrade A., Saatkamp T., Jiang Z., Cassegrain S., Zhao N., Shi Z., Holdcroft S. (2023). Performance
and Stability of Aemion and Aemion+ Membranes in Zero-Gap CO_2_ Electrolyzers with Mild Anolyte Solutions. ChemSusChem.

[40] Disch J., Bohn L., Koch S., Schulz M., Han Y., Tengattini A., Helfen L., Breitwieser M., Vierrath S. (2022). High-Resolution Neutron Imaging of Salt Precipitation
and Water Transport in Zero-Gap CO_2_ Electrolysis. Nat. Commun..

[41] Li D., Liu T., Yan Z., Zhen L., Liu J., Wu J., Feng Y. (2020). MOF-Derived Cu_2_O/Cu Nanospheres Anchored
in Nitrogen-Doped
Hollow Porous Carbon Framework for Increasing the Selectivity and
Activity of Electrochemical CO_2_-to-Formate Conversion. ACS Appl. Mater. Interfaces.

[42] Xu L.-W., Qian S.-L., Dong B.-X., Feng L.-G., Li Z.-W. (2022). The Boosting
of Electrocatalytic CO_2_-to-CO Transformation by Using the
Carbon Nanotubes-Supported PCN-222­(Fe) Nanoparticles Composite. J. Mater. Sci..

[43] Corsino D. C., Balela M. D. L. (2017). Room Temperature
Sintering of Printer Silver Nanoparticle
Conductive Ink. IOP Conf. Ser.: Mater. Sci.
Eng..

[44] Chanda V., Blaudszun D., Hoof L., Sanjuán I., Pellumbi K., junge Puring K., Andronescu C., Apfel U.-P. (2024). Exploring the (Dis)-Similarities
of Half-Cell and Full
Cell Zero-Gap Electrolyzers for the CO_2_ Electroreduction. ChemElectroChem..

[45] Mardle P., Cassegrain S., Habibzadeh F., Shi Z., Holdcroft S. (2021). Carbonate
Ion Crossover in Zero-Gap, KOH Anolyte CO_2_ Electrolysis. J. Phys. Chem. C.

[46] Yuan S., Wang E., Xue R., Wu L., Zhang G., Li H., Wang Q., Yin J., Luo L., Shen S., An L., Yan X., Zhang J. (2024). Flow Field
Design Matters for High
Current Density Zero-Gap CO_2_ Electrolyzers. ACS Energy Lett..

[47] Biemolt J., Singh J., Prats Vergel G., Pelzer H. M., Burdyny T. (2025). Preventing
Salt Formation in Zero-Gap CO_2_ Electrolyzers by Quantifying
Cation Accumulation. ACS Energy Lett..

[48] Lin J., Zhang Y., Xu P., Chen L. (2023). CO_2_ Electrolysis:
Advances and Challenges in Electrocatalyst Engineering and Reactor
Design. Materials Reports: Energy.

