# Supplementary material for: A Nature-Inspired Solution for Water Management in a Zero-Gap CO2 Electrolyzer
Source: ACS Energy Lett. 2025 Jun 6;10(7):3081–8. doi: 10.1021/acsenergylett.5c01243 (PMC12261320; doi:10.1021/acsenergylett.5c01243)
Supplement: Supplementary file 1 [file nz5c01243_si_001.pdf]

Supporting information

A nature-inspired solution for water management in a zero-gap CO_2 electrolyzer

Linlin Xu [a,‡], Panagiotis Trogadas [a,b,‡,], Yang Lan [a], Shuxian Jiang [a], Shangwei Zhou [c], Francesco Iacoviello [c], Wenjia Du [d], Rhodri Jervis [c], and Marc-Olivier Coppens [a,*]*

[a] Centre for Nature-Inspired Engineering, Department of Chemical Engineering, University College London, London WC1E 7JE, United Kingdom

[b] Department of Chemistry, Aristotle University of Thessaloniki, Thessaloniki 54124, Greece

[c] Electrochemical Innovation Lab, Department of Chemical Engineering, University College London, London WC1E 7JE, United Kingdom

[d] Department of Engineering Science, University of Oxford, Oxford OX1 3PJ, United Kingdom

‡ Both authors contributed equally

Corresponding Authors

* m.coppens@ucl.ac.uk (Marc-Olivier Coppens), trogadas@chem.auth.gr (Panagiotis Trogadas)

Section S1. Experimental

S1.1 Materials

Potassium bicarbonate ($KHCO_3$, ≥ 99.5%), 2-propanol (≥ 99.8%), Nafion™ 117 solution (~ 5% in a mixture of lower aliphatic alcohols and water), and silver (Ag) nanopowder (< 100 nm particle size, 99.5% trace metals basis) were purchased from Sigma-Aldrich. Potassium hydroxide (KOH, ≥ 85%) was obtained from Fisher Chemical. Sigracet 39 BB carbon gas diffusion layer (GDL) and Sustainion® X37-50 Grade RT Membrane were acquired from Dioxide Materials™. Deionized (DI) water with a specific resistance of 18.2 MΩ cm was used for all solution preparations throughout the experiments.

S1.2 Electrode preparation and membrane pretreatment

To prepare the cathode GDE, Ag nanoparticles were dispersed in 2-propanol at a concentration of 5 mg mL^{-1} and mixed with 5 wt% Nafion™ 117 solution. This ink solution was homogenized in an ultrasonic bath for 25 min, with the bath temperature carefully maintained below 30 °C using ice chunks. The Ag dispersion was then sprayed onto the microporous layer of a Sigracet 39 BB GDL using a handheld airbrush (IWATA-HP-BCP). The catalyst loading of the cathode electrode was 1.0 ± 0.1 mg cm^{-2}. For the anode electrode, an IrO_x sintered porous titanium frit (250 μm thick) with a mass loading of 1.0 ± 0.1 mg cm^{-2} was used.

To activate the Sustainion® X37-50 Membrane, as detailed in the product manual provided by the supplier, it was first soaked in a 1 M KOH solution for 8 h to facilitate swelling and separation from the liner. The membrane was then further activated by soaking in a fresh 1 M KOH bath at room temperature for a minimum of 24 h. Before installation in the zero-gap CO_2 electrolyzer, the membrane was thoroughly washed with DI water to remove any residual KOH from the surface.

S1.3 Flow-field fabrication

The parallel and single-serpentine flow-fields in the cathode of a zero-gap CO_2 electrolyzer were fabricated from 6 mm thick graphite plates (Schunk) using a CNC machine (Roland 40A). The channels were designed with dimensions of 1 mm in width, spacing, and depth. For the lizard-inspired single-serpentine flow-field, a Compact Laser Micromachining System (Oxford Lasers) was used to engrave the interconnected capillary arrays on the surface of the single-serpentine flow-field. These capillary channels were approximately 70 μm in width, 150 μm in depth, and 500 μm in spacing. After laser engraving, the graphite surfaces were gently washed with a small amount of DI water and then dried using an air gun to remove any debris. The designs of the parallel, single-serpentine, and lizard-inspired single-serpentine flow-fields are shown in Figure S1.

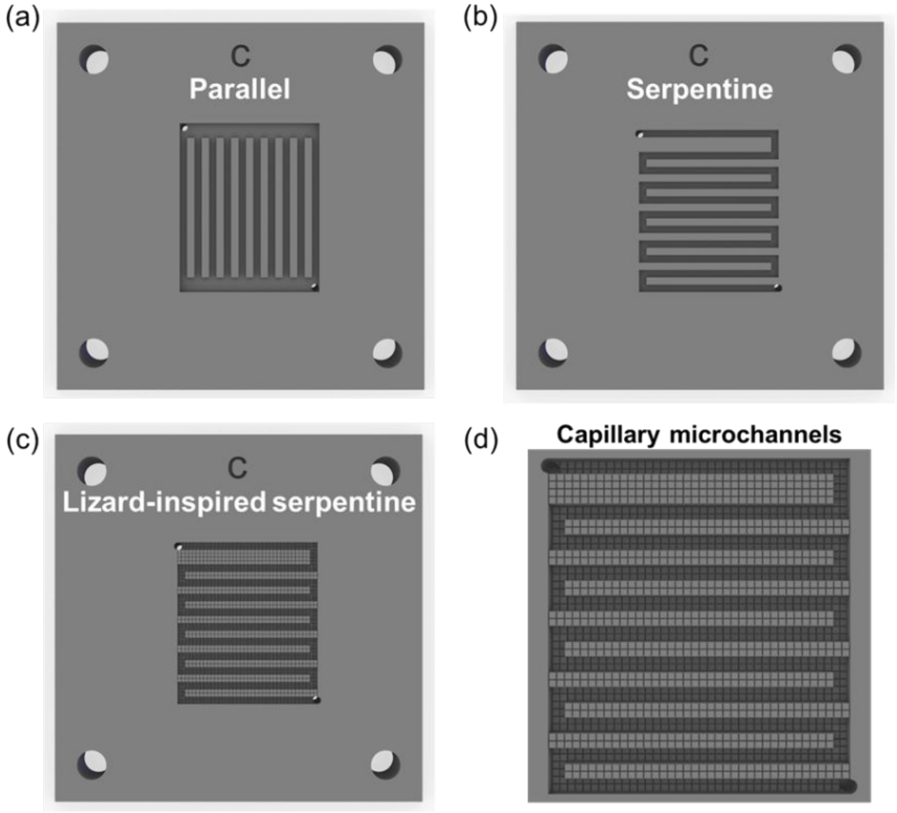

Figure S1. Illustration of the different flow patterns at the cathode: (a) parallel, (b) single-serpentine, (c) lizard-inspired serpentine, and (d) details of capillary microchannels on lizard-inspired serpentine flow-field.

S1.4 Zero-gap CO_2 electrolyzer components

All experiments were conducted in a custom-made zero-gap CO_2 electrolyzer (Figure S2), comprising the anode and cathode end plates, current collectors, flow-fields, gas diffusion electrodes and a membrane. The fully hydrated membrane was mounted between a cathode Ag GDE and an IrO_x titanium frit anode, with the catalyst layers facing the membrane. The Ag GDE had a geometric area of 2.25 cm^2. To secure the electrodes in position and control their

compression, two 0.24 mm thick PTFE gaskets were installed around each electrode. The cell assembly involved the use of eight bolt screws, each gradually tightened to a torque of 4 N m, ensuring optimal contact between the electrodes and the membrane, thereby facilitating effective sealing of the cell. A single-serpentine flow-field made of stainless steel was employed at the anode for all measurements due to its (electro)chemical stability in alkaline anolytes[1], while various graphite flow-field patterns (parallel, single-serpentine, and lizard-inspired single-serpentine) were used at the cathode.

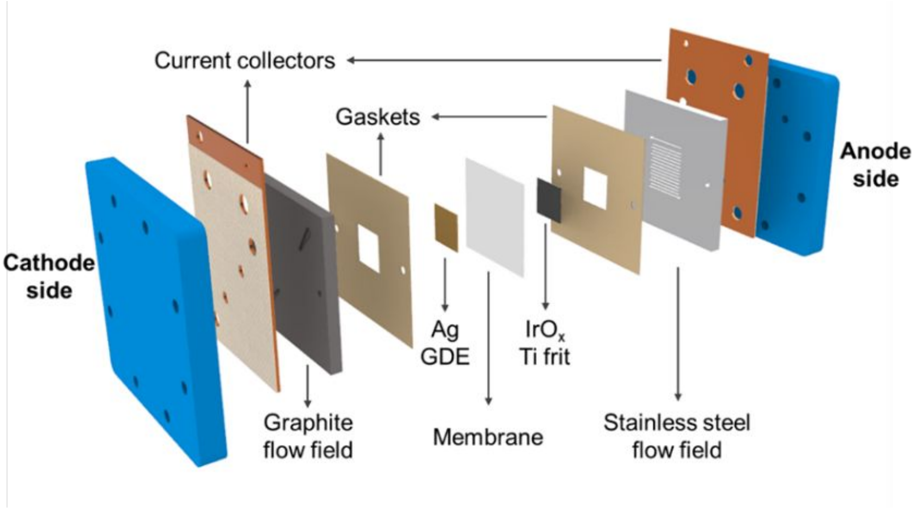

Figure S2. A zero-gap electrolyzer for CO_2RR.

S1.5 Set-up of the zero-gap CO_2 electrolyzer

As shown in Figure S3, the CO_2RR set-up on the cathode side began with the supply of constant gaseous CO_2 from a cylinder, which was directed into a mass flow meter (Bronkhorst® UK). The CO_2 (CP Grade, > 99.995%, BOC Gas) flow rate was set to 100 mL min^{-1}. Before reaching the cathode inlet, the CO_2 passed through a bubble bottle containing 100 mL of DI water. After the

electrochemical reaction, the resulting product gas stream was directed into a cooling condenser bottle, which removed water vapor from the gas stream before it was sent to the gas chromatograph (GC) for real-time gas composition analysis. On the anode side, the anolyte tank was filled with 200 mL of 0.1 M $KHCO_3$ solution. The anolyte was continuously delivered to the anode inlet of the electrolyzer by a peristaltic pump (Watson-Marlow Limited) operating at a flow rate of 20 mL min^{-1}. After circulating through the anode, the anolyte was returned to the bottle, ensuring a continuous supply for the electrochemical process.

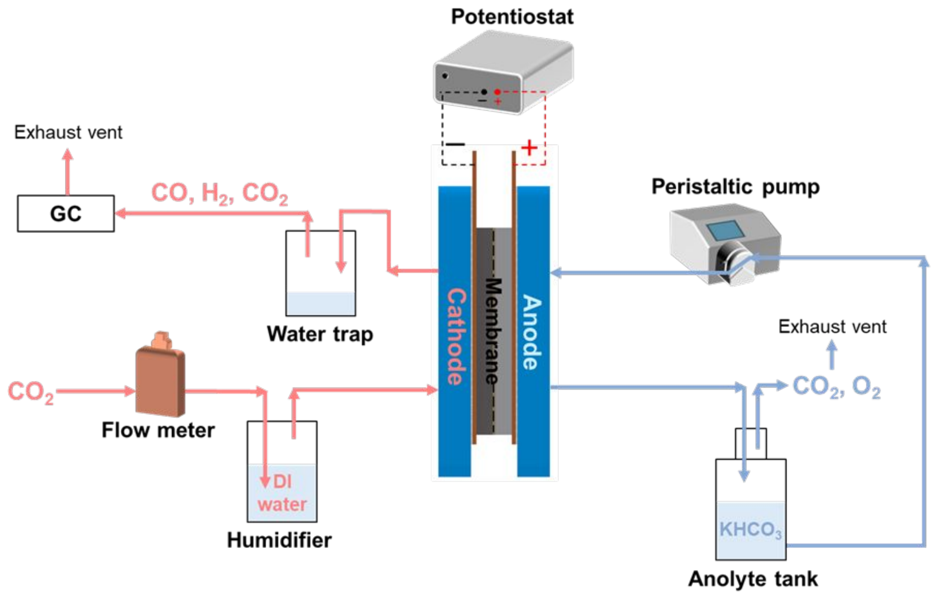

Figure S3. Flow diagram of the experimental setup used for CO_2RR.

S1.6 Electrochemical testing

The experimental setup for all measurements involved a two-electrode configuration with a Gamry Reference 3000 potentiostat (Gamry Instruments) operating in galvanostatic mode. Electrochemical impedance spectroscopy (EIS) was recorded over a frequency range of 100 kHz

to 0.1 Hz with an AC amplitude of 5% of the DC value. No IR correction was applied to the voltage values recorded during the experiments.

S1.7 Product analysis

The gas product composition was analyzed using a Shimadzu GC-2010 Pro gas chromatograph (Shimadzu UK Limited) equipped with a barrier discharge ionization detector (BID) and CP grade Helium carrier gas (> 99.999%, BOC Gas) at 10 min intervals. The external standard method was used for quantitative calculations.

Faradaic efficiency (FE) represents the fraction of the charge transferred to the product of interest compared to the total charge used in the electrochemical reaction:[2]

$$FE_i(\%) = \frac{nFC_i vP}{RTI} \quad (1)$$

where n denotes the number of electrons transferred to a product molecule, F is the Faraday constant (96485 C mol^{-1}), C_i represents the volume concentration of species i as measured by GC (%), v is the gas flow rate (m^3 s^{-1}), P is the atmospheric pressure (101325 Pa), R is the ideal gas constant (8.314 J mol^{-1} K^{-1}), T is the room temperature (K), and I is the applied current (A).

Partial current density (j_i) is the current density corresponding to a specific product.[3]

$$j_i = j_{total} \times FE_i(\%) \quad (2)$$

where j_{total} is the total applied current density (mA cm^{-2}).

Energy efficiency (EE_i) compares the cell voltage required to drive the reaction at a desired current density to the thermodynamic cell voltage.[4]

$$EE_i(\%) = \frac{E_0}{E_{cell}} \times FE_i(\%) \quad (3)$$

where $E_0 = -1.34\ V$ is the thermodynamic voltage for the electroreduction of CO_2 to CO coupled with the O_2 evolution reaction, and E_{cell} is the applied cell voltage (V).

S1.8 Sample characterization

Scanning electron microscopy (SEM) and energy-dispersive X-ray spectroscopy (EDS). The field emission SEM images were acquired with an EVO MA10 (Carl Zeiss) equipped with an EDS detector to examine the morphology and elemental distribution of the electrodes. The microscope was operated at 15 kV acceleration voltage.

X-ray diffraction (XRD) measurements. The powder XRD patterns were recorded in the scanning range (2θ) of 2-40° by a Stoe STADI-P diffractometer in transmission mode with monochromated Mo Kα1 radiation (wavelength λ = 0.70930 Å) and a Mythen detector (Dectris). Measurements were conducted with a step size of 0.5° and a duration of 5.0 s per step, achieving a resolution of 0.015°.

X-ray micro-computed tomography (Micro-CT). X-ray tomographic image acquisition of the GDE was performed using a ZEISS Xradia Versa 620 (Carl Zeiss, Dublin, CA, USA) instrument. All samples were scanned with a source voltage of 100 kV with exposure time of 1 s per projection, acquiring 2001 projections per scan. The raw transmission images from the scan were reconstructed using Reconstructor Scout-and-Scan software (Carl Zeiss, Dublin, CA, USA), which employs a cone-beam filtered back-projection algorithm. This process resulted in an isotropic voxel volume of approximately 1.78 μm.

Micro-CT image post-processing. For all reconstructed volumes, the material segmentation and post-processing were performed using Avizo Lite® (Thermo Fisher Scientific, USA). For each sample, a field-of-view of 300 voxel × 300 voxel × 200 voxel in x-y-z orientation was chosen for material segmentation. Since the signals of the metal components (Ag and K), are significantly more intensive than those of the other components and difficult to differentiate, each sample is segmented into three parts, namely metal, carbon and binder, and pores. The metal component represents Ag for the unreacted GDE, while it represents Ag and K for the reacted GDEs.

Section S2. Supporting figures

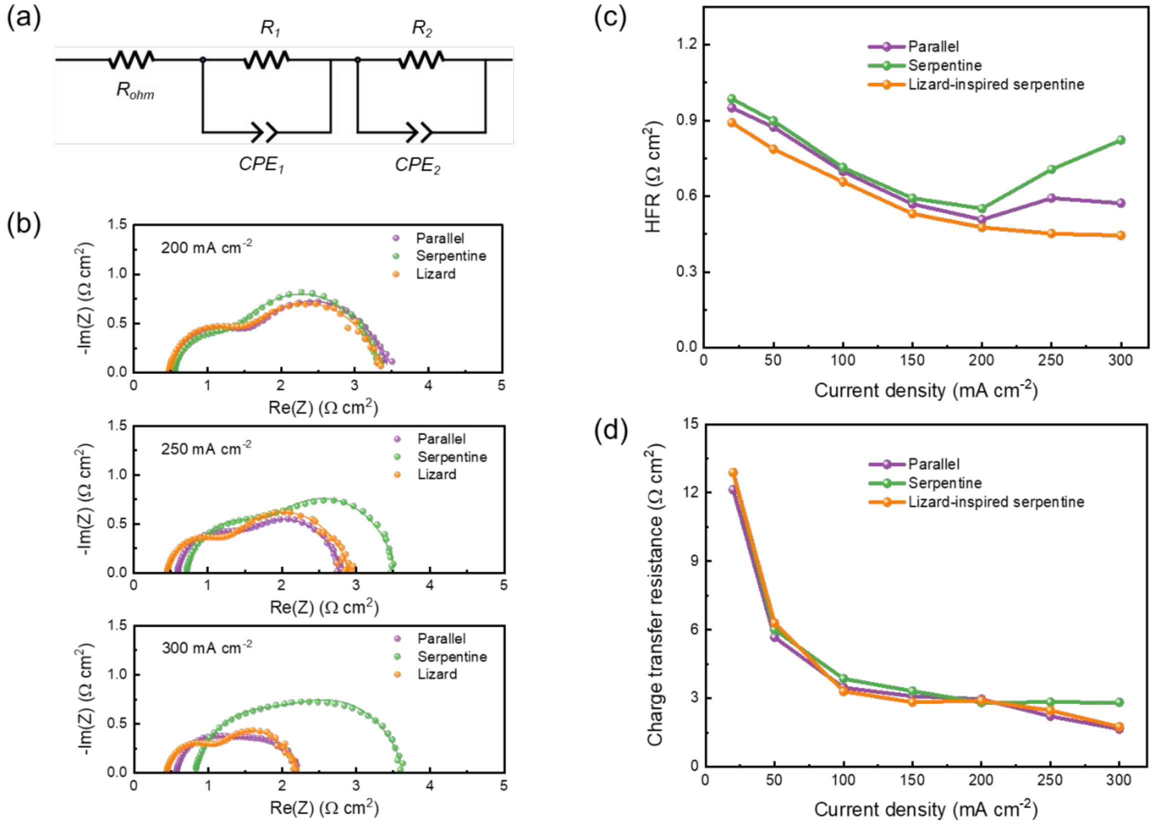

Figure S4. (a) Equivalent circuit model used for fitting of the EIS data. (b) Representative Nyquist plots at 200 mA cm^{-2}, 250 mA cm^{-2}, and 300 mA cm^{-2}. The solid lines are fitted results. Changes in (c) HFR and (d) the total charge transfer resistance with respect to current density. The HFR = R_{ohm}, while the total charge transfer resistance = R_1 + R_2. Data in (c) and (d) were collected at discrete current densities (20, 50, 100, 150, 200, 250, and 300 mA cm^{-2}). The geometric area of each flow-field is 2.25 cm^2.

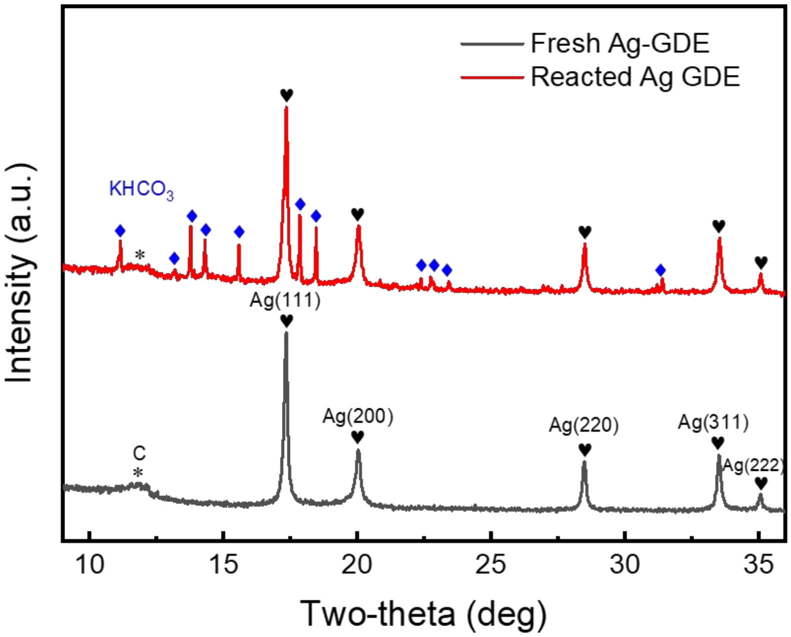

Figure S5. XRD pattern of Ag catalyst before and after CO_2RR. Based on the equal d-values in the Bragg equation, the diffraction angles of Ag and $KHCO_3$ with Mo Kα1 radiation in the figure were determined from the characteristic positions of Cu Kα1 radiation found in the literature.[5,6]

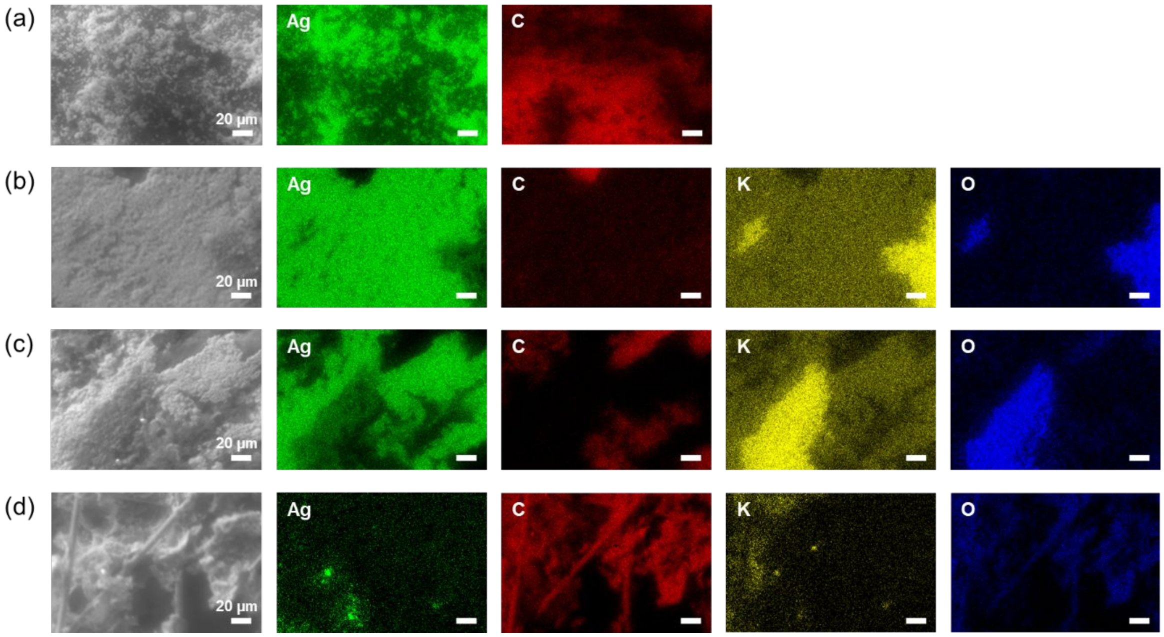

Figure S6. SEM images and EDS mapping of Ag GDEs (a) before and after electrolysis at 200 mA cm^{-2} with (b) parallel, (c) serpentine, and (d) lizard-inspired serpentine flow-field based CO_2 electrolyzers.

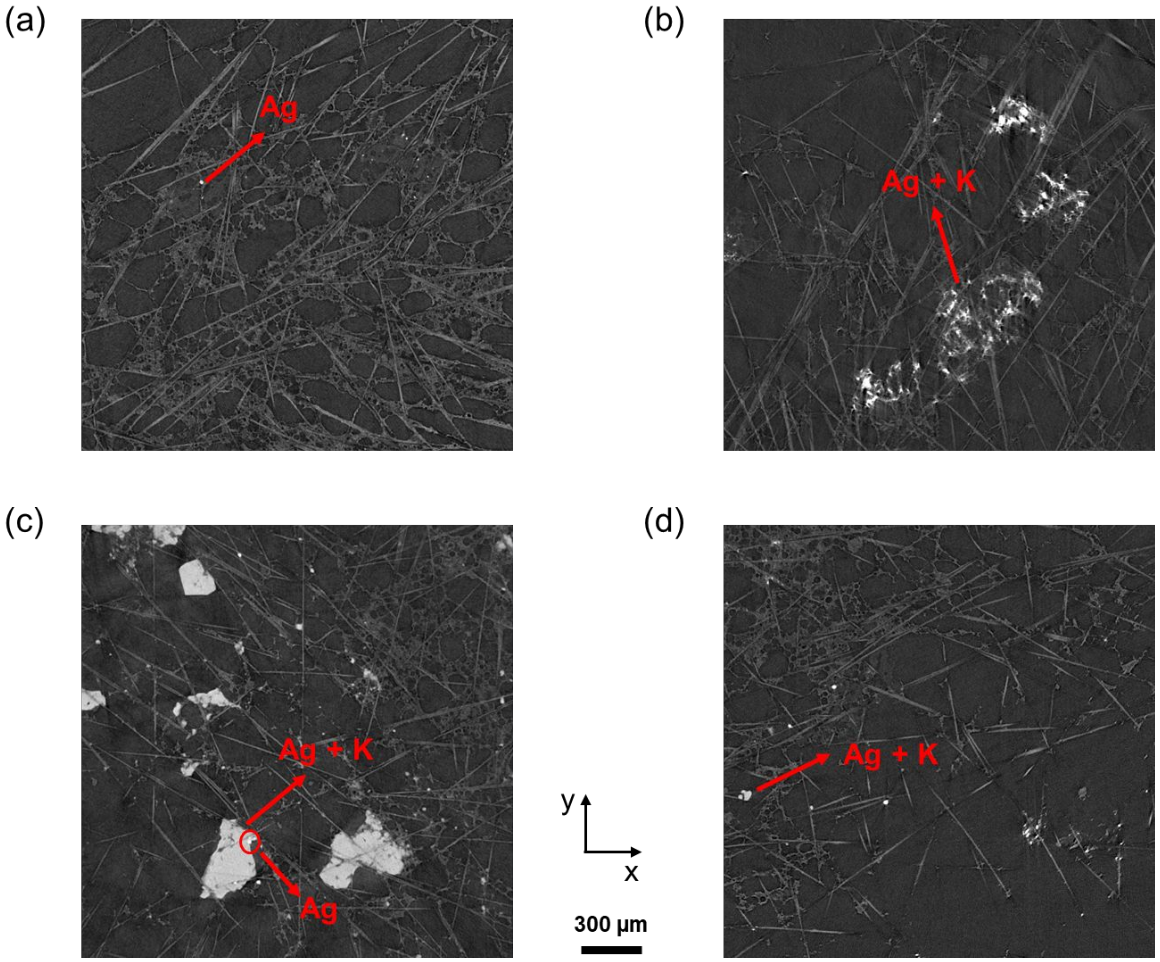

Figure S7. XY (top-down) view of micro-CT images of GDLs in cathode Ag GDEs (a) before, and (b-d) after electrolysis at 200 mA cm^{-2} with the (b) parallel, (c) serpentine, and (d) lizard-inspired serpentine flow-fields.

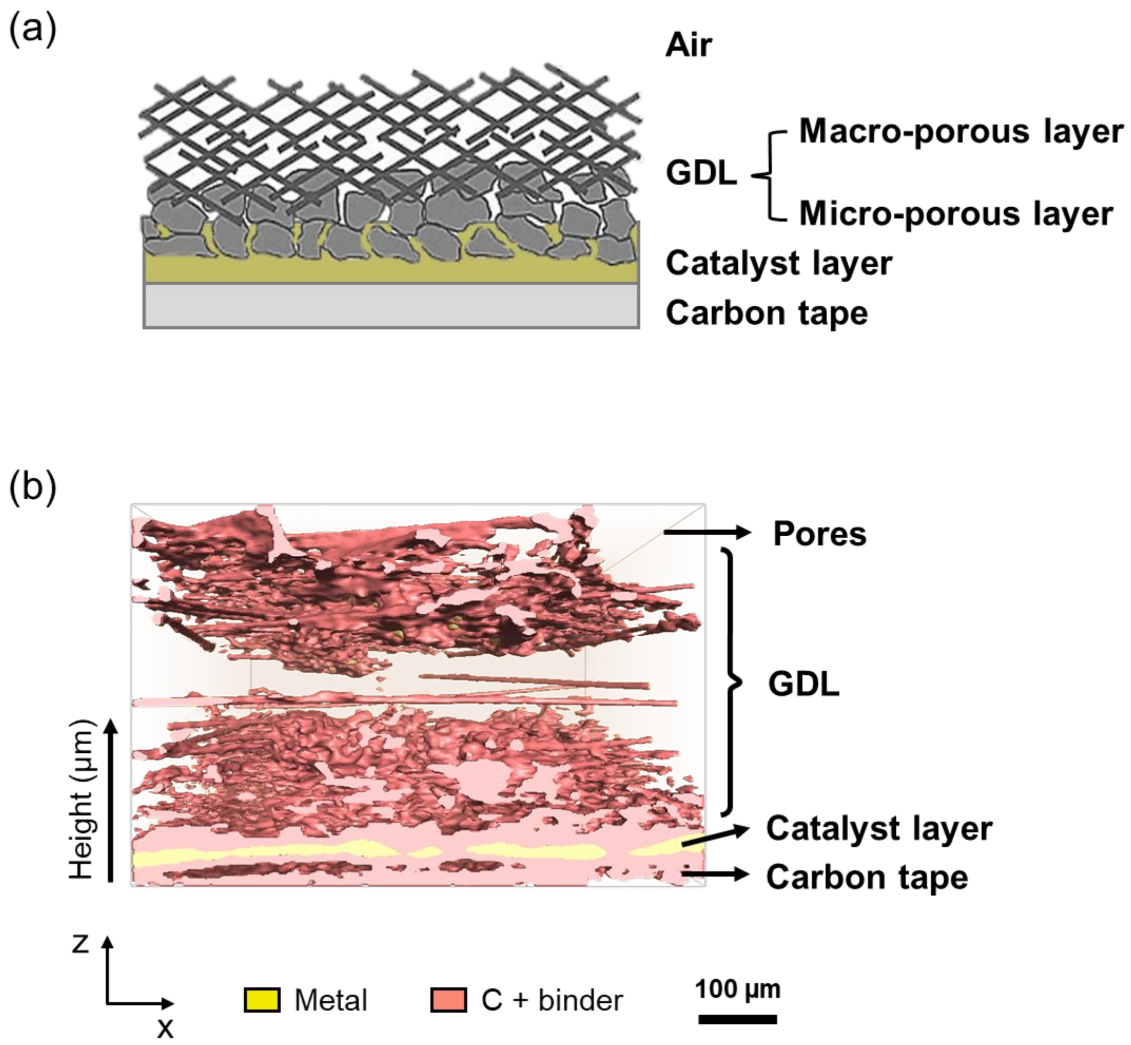

Figure S8. (a) The schematic diagram of samples, (b) an example of material segmentation of cathode Ag GDEs after electrolysis at 200 mA cm^{-2} with the parallel flow-field based CO_2 electrolyzer. “Height” in Figure S7b corresponds to the horizontal axis in Figure 5b.

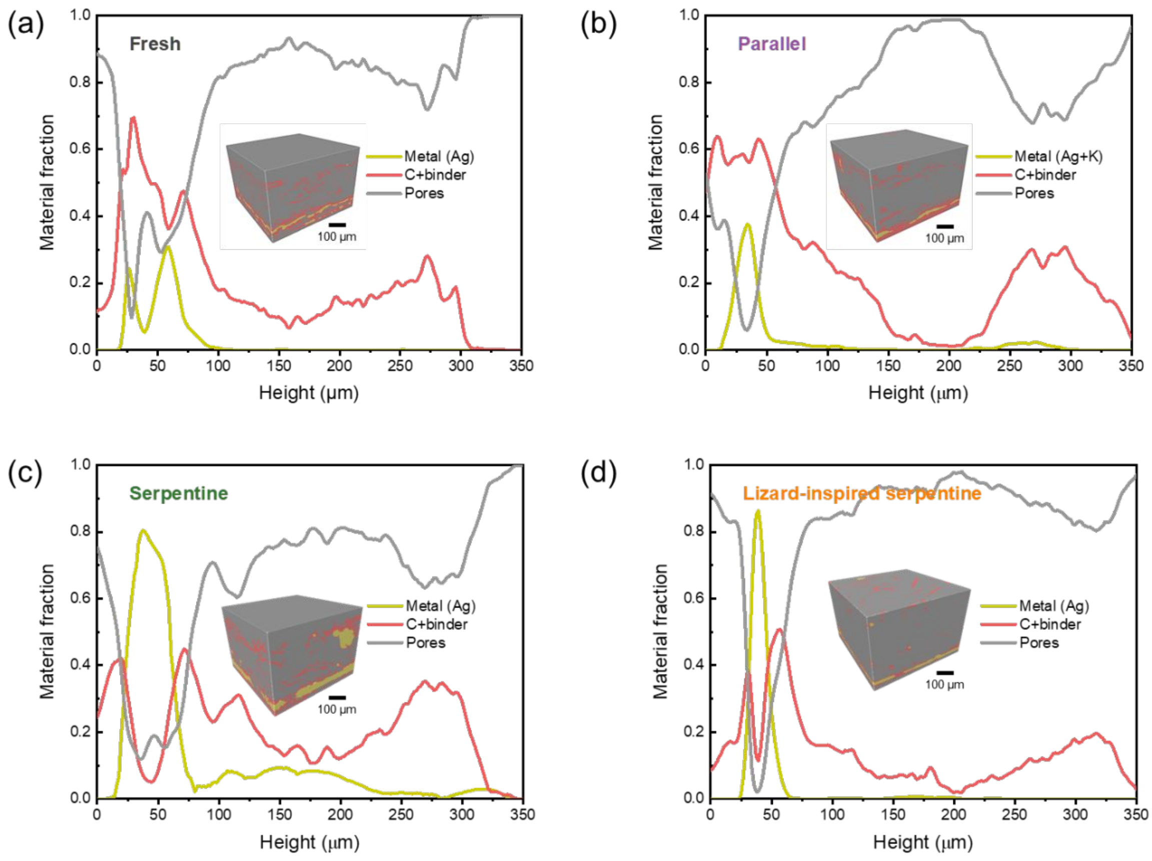

Figure S9. Material fraction of cathode Ag GDEs (a) before and after electrolysis at 200 mA cm^{-2} with (b) parallel, (c) serpentine, and (d) lizard-inspired serpentine flow-field based CO_2 electrolyzers. Inset images are the corresponding 3D material segmentations.

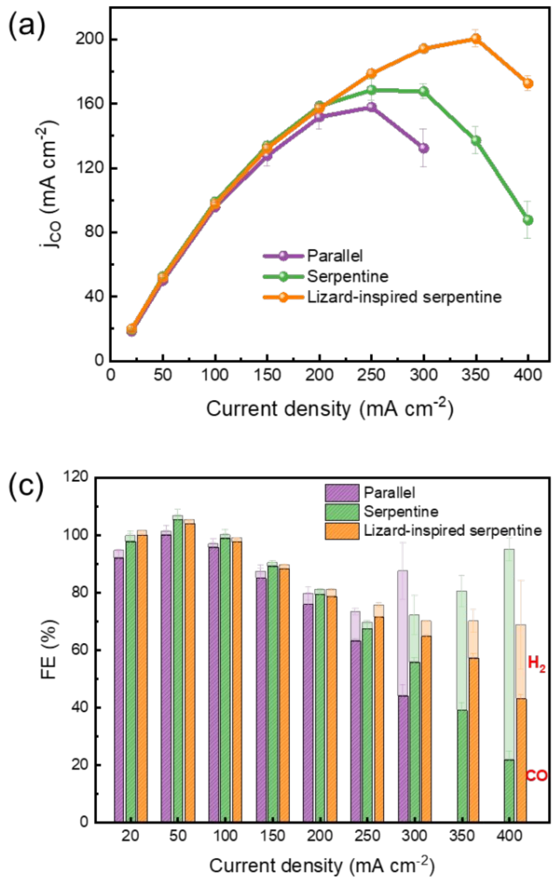

Figure S10. The average partial current density of (a) CO and H_2, (c) total Faradaic efficiency (CO-bottom bar, H_2-top bar), and (d) cell voltage for parallel, serpentine, and lizard-inspired serpentine flow-field based CO_2 electrolyzers. Data were collected at discrete current densities (20, 50, 100, 150, 200, 250, 300, 350 and 400 mA cm^{-2}). Each experiment is replicated across three independent measurements, and the error bars represent the standard deviation. The geometric area of each flow-field is 5.06 cm^2.

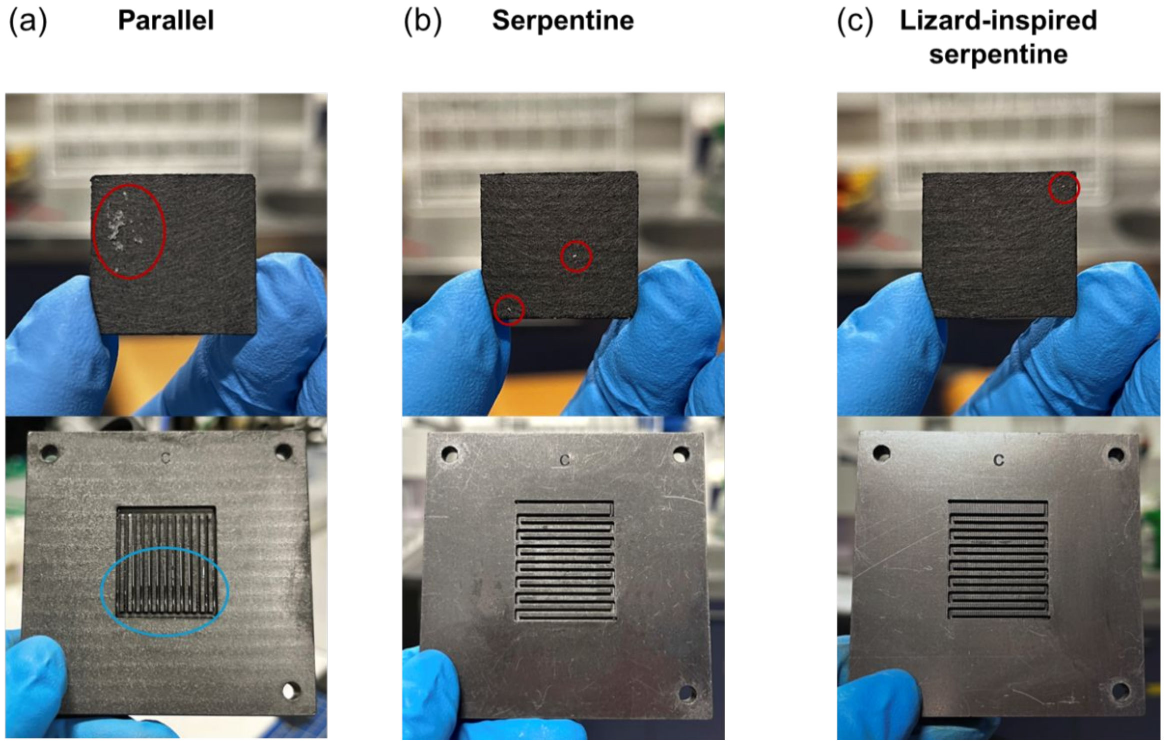

Figure S11. Images of the back of cathode Ag GDEs and cathode flow-fields after electrolysis for (a) parallel, (b) serpentine, and (c) lizard-inspired serpentine flow-field based CO_2 electrolyzers. Salt crystal precipitates are indicated in the red ellipse, while liquid water is shown in the blue ellipse.

References

[S1] Vennekötter, J.-B.; Scheuermann, T.; Sengpiel, R.; Wessling, M. The Electrolyte Matters: Stable Systems for High Rate Electrochemical CO_2 Reduction. *J. CO_2 Util.* **2019**, *32*, 202–213. https://doi.org/10.1016/j.jcou.2019.04.007.

[S2] Dutta, N.; Bagchi, D.; Chawla, G.; Peter, S. C. A Guideline to Determine Faradaic Efficiency in Electrochemical CO_2 Reduction. *ACS Energy Lett.* **2024**, *9* (1), 323–328. https://doi.org/10.1021/acsenergylett.3c02362.

[S3] Zhang, X.; Guo, S.-X.; Gandionco, K. A.; Bond, A. M.; Zhang, J. Electrocatalytic Carbon Dioxide Reduction: From Fundamental Principles to Catalyst Design. *Mater. Today Adv.* **2020**, *7*, 100074. https://doi.org/10.1016/j.mtadv.2020.100074.

[S4] Seong, H.; Choi, M.; Park, S.; Kim, H.; Kim, J.; Kim, W.; Yoo, J. S.; Lee, D. Promoting CO_2-to-CO Electroreduction via the Active-Site Engineering of Atomically Precise Silver Nanoclusters. *ACS Energy Lett.* **2022**, *7* (12), 4177–4184. https://doi.org/10.1021/acsenergylett.2c02018.

[S5] Corsino, D. C.; Balela, M. D. L. Room Temperature Sintering of Printer Silver Nanoparticle Conductive Ink. *IOP Conf. Ser.: Mater. Sci. Eng.* **2017**, *264*, 012020. https://doi.org/10.1088/1757-899X/264/1/012020.

[S6] Liu, A.; Lu, X.; Zhou, X.; Xu, C.; Liang, X.; Xiong, K. Experimental Investigation on Suppression of Methane Explosion Using $KHCO_3$/Zeolite Composite Powder. *Powder Technol.* **2023**, *415*, 118157. https://doi.org/10.1016/j.powtec.2022.118157.
